# *Staphylococcus argenteus* ST2250 from diabetic foot sepsis: genomic insights into resistance and virulence

**DOI:** 10.3389/fcimb.2026.1822021

**Published:** 2026-04-29

**Authors:** Hongdong Zhao, Chi Zhu, Shengxin Ye, Zhimin Bai, Zhiqiang Liu, Xin Dong, Yirong Zhang, Huizhen Chen, Jinjing Wu, Yinfeng Su, Yang Wang, Kaizhen Wen

**Affiliations:** 1 Department of Clinical Laboratory, Jinjiang Municipal Hospital (Shanghai Sixth People’s Hospital Fujian), Jinjiang, Fujian, China; 2 Department of Clinical Laboratory, Hangzhou Adicon Medical Laboratory Co., Ltd., Hangzhou, China

**Keywords:** antimicrobial resistance, diabetic foot infection, sepsis, ST2250, *Staphylococcus argenteus*, virulence genes, whole-genome sequencing

## Abstract

**Background:**

*Staphylococcus argenteus* ST2250 is a globally disseminated clone, but clinical data on its life-threatening infections in high-risk populations and molecular dissemination evidence remain limited.

**Methods:**

We integrated clinical data, antimicrobial susceptibility testing, and whole-genome sequencing (WGS) for four *S. argenteus* isolates—including paired blood and wound isolates from a septic shock case—comparatively analyzed against 370 global genomes. Clonal relatedness was assessed by core-genome SNP analysis, with resistance and virulence genes.

**Results:**

A 53-year-old uncontrolled type 2 diabetes patient developed septic shock with pronounced inflammatory response (PCT 13.32 ng/mL, CRP 364.85 mg/L). WGS confirmed 0 core-genome SNP differences between blood (20250131) and wound (20250137) isolates, validating clonal transmission from the foot ulcer. All four institutional isolates were ST2250, sharing 12 core antimicrobial resistance-associated (AMR) genes. Global analysis identified the dominance of ST2250 (55.9% of 370 genomes), with 101 out of 147 (68.7%) observed between 2015 and 2019 among the dated genomes (68.7% of 268 genomes with a known collection year). ST2250 exhibited unique AMR signatures: highest *blaZ* prevalence (66.7%), near-universal *fosB* (98.5%), and unique *tet(L)* (42.5%) and *APH(3’)-IIIa* (48.8%) carriage absent in ST2198, ST2793, ST2854, and ST5961. Virulence profiling revealed ST2250-specific features: significantly reduced clfB (11.1% vs. 87.5–100% in other STs, P<0.01), complete absence of *set23* and ESAT-6 system components (0% vs. 100%), and near-universal conservation of *cap8* operon (98.6%), *lukF-PV* (98.6%), and set25/26 (98.6%).

**Conclusions:**

This study provides genetic validation of *S. argenteus* ST2250-induced septic shock originating from diabetic foot infection. The clone’s global dominance, distinctive resistance profile, and reconfigured virulence architecture highlight the urgent need for enhanced diagnostic stewardship, targeted surveillance in diabetic populations, and rational antimicrobial use.

## 1 Introduction

*Staphylococcus argenteus* (*S. argenteus*) was formally recognized as a distinct species within the *Staphylococcus aureus* (*S. aureus*) complex in 2015, distinguished by phylogenomic divergence and absence of the *crtOPQMN* gene - required for staphyloxanthin biosynthesis - resulting in non-pigmented colonies (Tong et al., 2015; Becker et al., 2019). Among its diverse sequence types, ST2250 has emerged as a globally disseminated clone, with high prevalence in Asia and reports from both clinical infections (e.g., skin/soft tissue infections, bacteremia) and food sources (raw chicken, retail meat) (Suzuki et al., 2017; Meijer et al., 2022). Unique genetic features such as a type III-A CRISPR-Cas system (limiting horizontal gene transfer) and multiple enterotoxin genes contribute to its pathogenicity and spread (Chen et al., 2025), yet *S. argenteus* remains underrecognized clinically due to phenotypic similarities with *S. aureus* and outdated diagnostic databases (Zhang et al., 2016).

The clinical significance of *S. argenteus* is increasingly evident, with reports of invasive diseases including bacteremia, endocarditis, and septic shock (Rigaill et al., 2018; Goswami et al., 2021). Its pathogenic potential remains undiminished despite the loss of staphyloxanthin—an antioxidant and immune evasion factor in *S. aureus* (Thaipadungpanit et al., 2015; Chen et al., 2018), as it harbors multiple enterotoxin genes and a comprehensive repertoire of *S. aureus*-like virulence factors, enabling severe systemic inflammatory responses (Lee et al., 2024). However, several critical research gaps persist: data on ST2250-induced life-threatening infections in diabetic patients—who are inherently predisposed to foot ulcers and invasive bacterial infections—remain scarce, and while sporadic *S. argenteus* bacteremia cases have been documented, molecular evidence confirming clonal homology between primary infection foci and bloodstream isolates is rare, leaving dissemination pathways poorly defined (Fang et al., 2024; Gatti et al., 2024). Furthermore, disparities in antimicrobial resistance-associated (AMR) and virulence genes between ST2250 and other *S. argenteus* sequence types have not been clearly delineated, hindering targeted prevention and treatment strategies.

Herein, we report a case of septic shock secondary to a diabetic foot infection caused by *S. argenteus* ST2250, integrating detailed clinical data, antimicrobial susceptibility profiling, and whole-genome sequencing (WGS) of institutional isolates. Comparative genomics with global *S. argenteus* collections elucidates the clone’s pathogenic potential (AMR and virulence genes) and molecular epidemiology. The study aimed to validate the clonal link between the diabetic foot ulcer and bloodstream infection, clarify ST2250’s global molecular epidemiology, delineate its unique AMR and virulence gene profiles, and ultimately inform infection control, diagnostic stewardship, and surveillance strategies for this underrecognized pathogen in high-risk populations.

## 2 Methods

### 2.1 Clinical case description and sample collection

A 53-year-old male with poorly controlled type 2 diabetes (HbA1c 16.6%; admission blood glucose >27.8 mmol/L) presented to our institution on February 25, 2025, with 10–15 days of fatigue, anorexia, drowsiness, and a ruptured, swollen right lateral malleolar ulcer (no trauma history). Initial vital signs: blood pressure 74/47 mmHg, heart rate 105 bpm, temperature 36.2 °C. Laboratory results: white blood cell count 28.67*10^9^/L (neutrophils 27.24*10^9^/L, 95%), C-reactive protein (CRP) 364.85 mg/L, procalcitonin (PCT) 13.32 ng/mL, creatinine 187.7 μmol/L, estimated glomerular filtration rate (eGFR) 32.6 mL/min, pH 7.00, blood glucose >27.8 mmol/L, pro-BNP 2941 pg/ml, glomerular filtration rate: 37 ml/min/1.73 m^2^, and potassium 3.30 mmol/L. The initial clinical diagnosis encompassed septic shock, foot tissue infection, diabetic ketoacidosis, acute kidney injury, hypokalemia, and hypoproteinemia.

Upon admission (18:00, February 25), four blood culture sets (two aerobic and two anaerobic bottles) were collected and processed using the BACTEC FX automated system (BD Diagnostics, Franklin Lakes, NJ, USA). Wound exudate was simultaneously sampled from the foot ulcer using sterile swabs. Retrospective review of our institution’s microbiology archives (January 2023–February 2025) identified two additional *S. argenteus* isolates (20230185 and 20250208) typed by matrix-assisted laser desorption/ionization time-of-flight mass spectrometry (MALDI-TOF MS).

### 2.2 Microbiological investigations

Positive blood culture bottles and wound specimens were subcultured onto blood agar and incubated at 37 °C for 18–24 hours. Species identification was performed via MALDI-TOF MS (Bruker Microflex LT/SH, Bruker Daltonics, Bremen, Germany) using the MBT Compass Library 2023 Revision P. Antimicrobial susceptibility testing was conducted with the VITEK-2 Compact system (bioMérieux, Marcy-l’Étoile, France) per CLSI guidelines, with minimum inhibitory concentrations (MICs) determined for 15 agents: penicillin G, oxacillin, erythromycin, teicoplanin, clindamycin, gentamicin, vancomycin, moxifloxacin, levofloxacin, linezolid, trimethoprim/sulfamethoxazole, rifampin, daptomycin, tigecycline, and β-lactamase detection. Notably, fosfomycin and tetracycline was not included in the standard VITEK-2 panel for blood culture isolates.

### 2.3 Whole-genome sequencing and species confirmation

Genomic DNA was extracted from clinical isolates and subjected to WGS using the DNBSEQ-T7RS platform (MGI Tech, Shenzhen, China). Raw reads were quality-controlled with fastp v0.24.0 (adapter trimming, removal of ambiguous/low-quality reads) (Chen, 2023). Species composition was assessed via Kraken2 v2.1.3 with Bracken v2.9 post-processing utilizing the Standard database (Lu et al., 2017; Wood et al., 2019). High-quality reads were *de novo* assembled using SPAdes v4.0.0 with default parameters. Genome completeness and contamination were evaluated using CheckM2 v1.02. Average Nucleotide Identity (ANI) was computed using skani v0.3.0 against the *S. argenteus* reference genome SP2 (GCA_000236925.1) (Shaw and Yu, 2023). Multilocus sequence typing (MLST) was performed with mlst v2.22.0 (https://github.com/tseemann/mlst) employing the S. aureus PubMLST schema.

For clonal relatedness, Core-genome single nucleotide polymorphism (SNP) analysis was conducted using Snippy v4.6.0 (https://github.com/tseemann/snippy, reference: GCA_000236925.1), with recombination regions removed by Gubbins v2.4.1 and SNP distance matrices generated by snp-dists v0.8.2 (Croucher et al., 2015).

### 2.4 Comparative genomics and phylogenetic reconstruction

For comparative phylogenomics, a global collection of 468 *S. argenteus* genomes was retrieved from NCBI GenBank. Quality filtering was applied to ensure genomic integrity: genomes were retained if they met the following criteria: (i) Average Nucleotide Identity (ANI) ≥95% relative to the *S. argenteus* reference genome SP2 (GCA_000236925.1); (ii) completeness ≥95% as assessed by CheckM2; and (iii) contamination ≤5%. Four institutional isolates were phylogenetically integrated with the global dataset. Maximum likelihood phylogeny was inferred using IQ-TREE 2 v2.4.0 (Minh et al., 2020), with trees annotated and visualized via ggtree v3.10.1 (Xu et al., 2022).

### 2.5 Resistance and virulence gene comparison

Antimicrobial resistance genes were annotated using ABRicate v1.0.1 against the Comprehensive Antibiotic Resistance Database (CARD v4.0.1), and virulence genes were identified against the Virulence Factor Database (VFDB v2025.11.07), retaining hits with >90% coverage and identity in both analyses (Alcock et al., 2023; Zhou et al., 2025). Heat maps were utilized to compare variations in drug resistance and virulence across bacterial strains.

### 2.6 Statistical analysis

Comparisons of categorical variables (virulence and resistance-associated gene carriage rates) between sequence types were performed using Pearson’s chi-square test or Fisher’s exact test (when expected cell frequencies were <5) using R version 4.3.1 (R Foundation for Statistical Computing, Vienna, Austria).

For analyses involving >2 STs, the Benjamini-Hochberg procedure was applied to control the false discovery rate (FDR). Adjusted P-values (q-values) <0.05 were considered significant.

## 3 Results

### 3.1 Clinical presentation and therapeutic outcomes

Empirical therapy with meropenem (1g q12h) was initiated on February 25, with vancomycin (0.5g qd) added on February 26 following culture results. Both antimicrobials were continued until March 2. Supportive care included fluid resuscitation, insulin infusion, potassium supplementation, and albumin infusion. Clinical improvement was evidenced by declining leukocytosis (28.67 to 18.64×10^9^/L), CRP (364.85 to 119.39 mg/L), and PCT (13.32 to 4.51 ng/mL) over 6 days. The patient was discharged on March 2, 2025, at the family’s request.

### 3.2 Phenotypic characterization of clinical isolates

**Figure 1:**
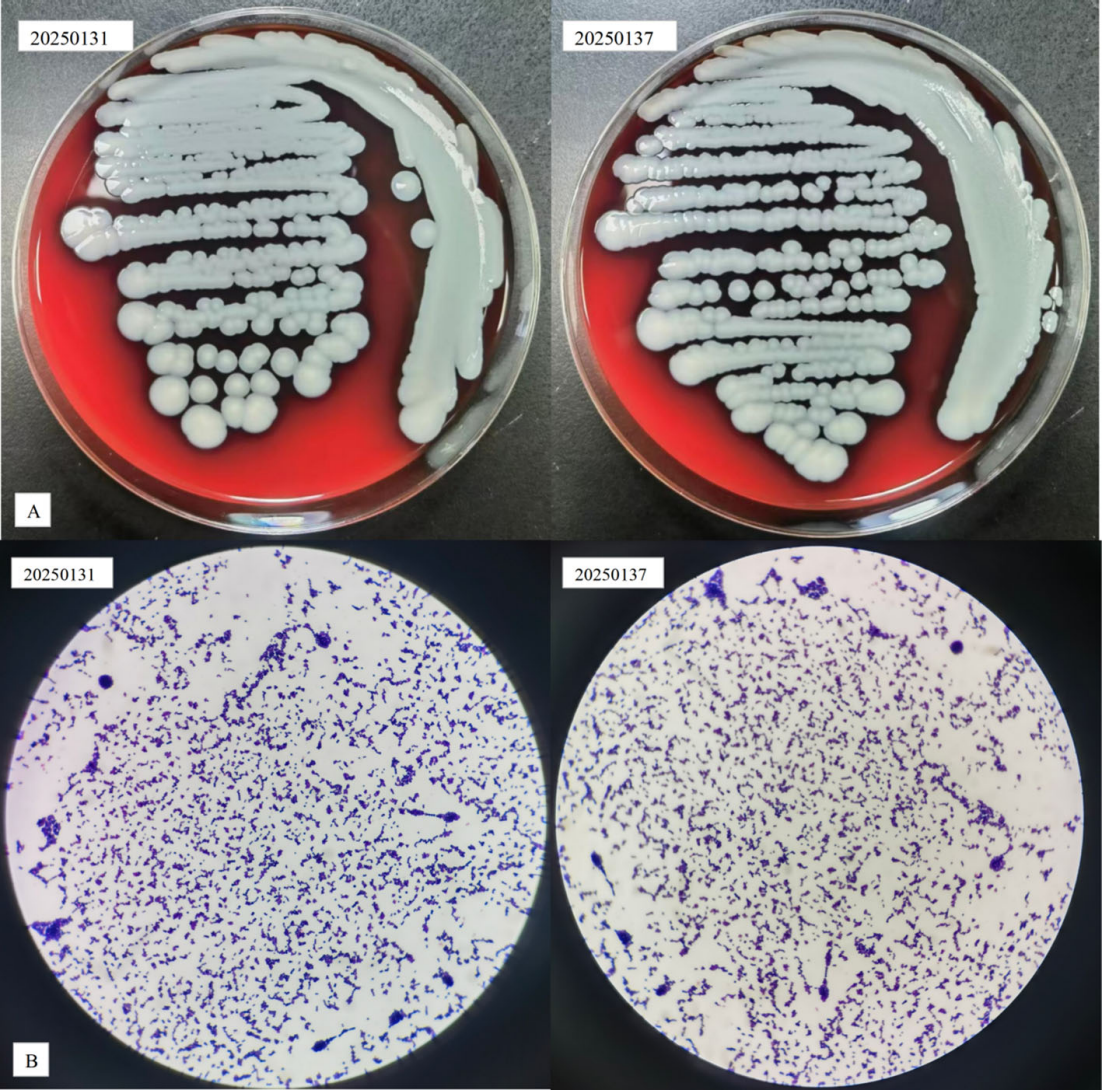
Colony morphology and Gram staining of *S. argenteus* isolates. **(A)** Colony morphology of isolates 20250137 (foot exudate) and 20250131 (blood) on blood agar medium showing non-pigmented, creamy colonies. **(B)** Gram staining demonstrating Gram-positive cocci in clusters.

All four blood culture bottles were positive within 9.6–11.04 hours (mean: 10.12 hours). MALDI-TOF MS identified foot exudate (20250137) and blood (20250131) isolates as *S. argenteus* (log scores 2.34 and 2.31, respectively). Colonies were non-pigmented and creamy on blood agar, with Gram-positive cocci in clusters (**Figure 1**). Antimicrobial susceptibility testing demonstrated pan-susceptibility to all 15 agents, including vancomycin (MIC 1 μg/mL), daptomycin (MIC 0.5 μg/mL), and linezolid (MIC 2 μg/mL), with negative β-lactamase activity (**Table 1**).

**Table 1:** Antimicrobial susceptibility profiles (MIC, μg/mL) of *S. argenteus* isolates.

Antimicrobial agent	MIC	Susceptibility
Penicillin G	0.06	Sensitive
Oxacillin	≤0.25	Sensitive
Erythromycin	≤0.25	Sensitive
Teicoplanin	≤0.5	Sensitive
Clindamycin	0.25	Sensitive
Gentamicin	≤0.5	Sensitive
Vancomycin	1	Sensitive
Moxifloxacin	≤0.25	Sensitive
Levofloxacin	0.25	Sensitive
Linezolid	2	Sensitive
Trimethoprim/Sulfamethoxazole	≤10	Sensitive
Rifampin	≤0.5	Sensitive
Daptomycin	0.5	Sensitive
Tigecycline	≤0.12	Sensitive
β-lactamase	/	Negative

Susceptibility testing was performed on index case isolates 20250131 (blood) and 20250137 (wound exudate), which showed identical MIC profiles.

### 3.3 Clonal identity and molecular epidemiology

WGS generated high-quality data for all isolates (Q30 >90%, coverage >500×). Kraken2/Bracken assigned >90% of reads to *S. argenteus* (TaxID: 985002), with 7-10% mapping to *S. aureus* (**Supplementary Table 1**). CheckM2 confirmed 100% genome completeness and <0.7% contamination (**Supplementary Table 2**). ANI between clinical isolates ranged from 99.87-99.89%, confirming conspecificity (**Supplementary Figure 1**).

**Figure 2:**
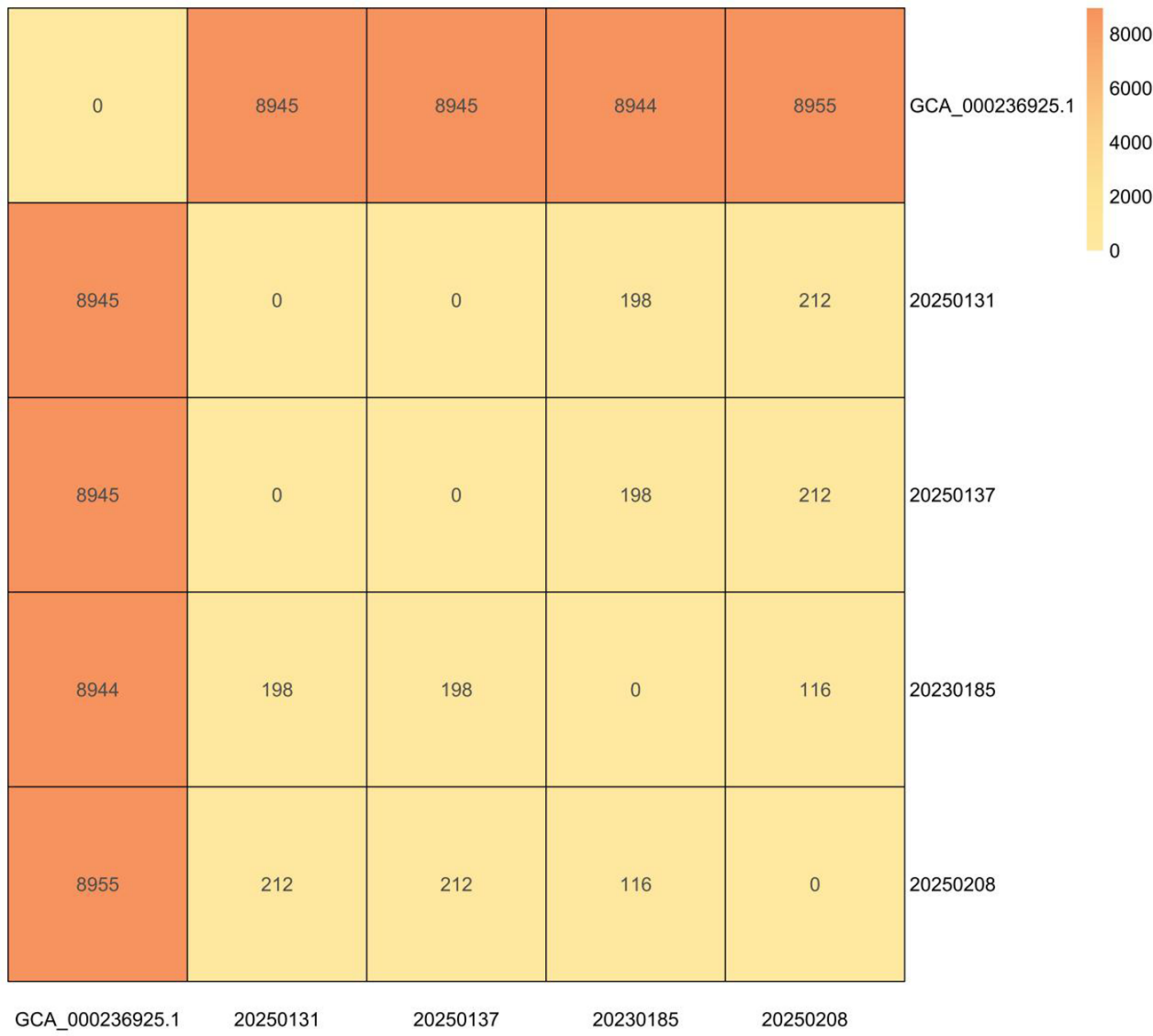
Core-genome SNP distance matrix of hospital S. argenteus ST2250 isolates. Heatmap shows pairwise SNP distances between the four institutional isolates and the reference genome (GCA_000236925.1). Core-genome SNP analysis performed using Snippy v4.6.0, with recombination regions removed by Gubbins v2.4.1 and distance matrix generated by snp-dists v0.8.2. Darker colors indicate greater genetic distance; 0 SNPs (white) between 20250131 and 20250137 confirms clonal identity.

Core-genome SNP analysis revealed 0 SNP differences between blood (20250131) and wound (20250137) isolates. Isolate 20250208 differed by 212 SNPs, while isolate 20230185 diverged by 198 SNPs (**Figure 2**).

### 3.4 Global phylogenetic context and geographic-temporal dynamics

For comparative phylogenomics, 468 *S. argenteus* genome assemblies were retrieved from the NCBI GenBank public database. These represented whole-genome sequencing data from cultured clinical, food-derived, and veterinary isolates submitted by independent research groups globally. Quality filtered by ANI ≥95%, completeness ≥95% and contamination ≤5%. All 468 genomes met ANI and completeness criteria; 98 genomes (20.9%) were excluded due to contamination >5%, yielding 370 high-quality genomes.

Temporal and geographic distribution of the 370 high-quality genomes are summarized in **Supplementary Table 3**, **Supplementary Figure 2**. Collection dates were available for 268 genomes (72.4%): 6/370 (1.6%) from 2005–2009, 56/370 (15.1%) from 2010–2014, 147/370 (39.7%) from 2015–2019, and 59/370 (15.9%) from 2020–2025; 102/370 (27.6%) lacked collection year metadata. ST2250 was the dominant sequence type overall (207/370, 55.9%), followed by ST1223 (46/370, 12.4%), ST2198 (24/370, 6.5%), and ST5961 (18/370, 4.9%). Among ST2250 isolates with available metadata, isolates with collection dates were most frequently reported during 2015–2019 (101/147, 68.7% of temporally annotated ST2250 genomes). ST2250 showed geographic variation: Thailand 64/77 (83.1%), non-Asian countries 62/106 (58.5%), China 15/44 (34.1%), Japan 6/22 (27.3%). Temporal distribution of ST2250 (**Supplementary Figure 2**): The highest number of reported isolates occurred in 2015 (Thailand: n=58; China: n=6) and 2018–2019 (non-Asian countries: n=27).

**Figure 3:**
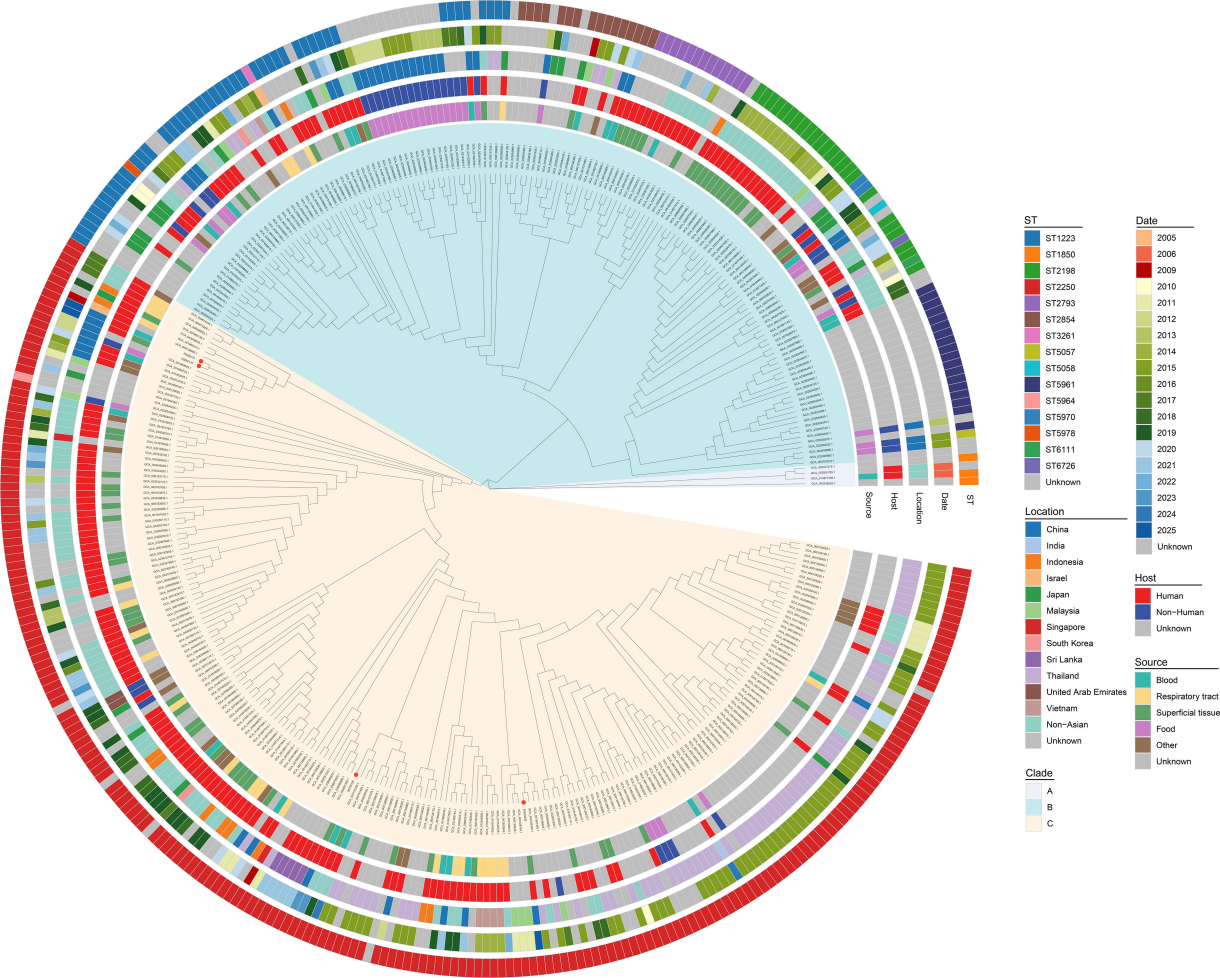
Maximum likelihood phylogenetic tree of 370 global *S. argenteus* isolates, colored by sequence type, host, source, location, and collection year.

Institutional isolates exhibited distinct geographic affinities: index case isolates (20250131/20250137) were closely related to 2012 Chinese food-derived isolates (98 SNPs to Ningbo isolate GCA_001969695.1; 106 SNPs to Shanghai pork isolate GCA_001969725.1). Isolate 20230185 clustered with non-Asian isolates (69 SNPs to Italian blood isolate GCA_033151035.1; 67 SNPs to Brazilian foot isolate GCA_026191495.1). Isolate 20250208 showed 80–86 SNPs to 2015 Thai isolates (GCA_900128345.1, GCA_900126825.1) (**Figure 3**).

### 3.5 Resistance-associated and virulence genomic profile

#### 3.5.1 Antimicrobial resistance-associated genes

Four institutional isolates shared a core AMR profile of 12 genes, encoding (i) regulatory genes (*mgrA*, *arlS/arlR*, *mepR/mepA*) that modulate efflux pump expression; (ii) efflux pump genes (*norA*, *LmrS*, *sdrM*, *sepA*, *norC*); and (iii) direct resistance determinants (*fosB*, *tet(38)*) (**Table 2**). Isolate 20250208 uniquely harbored *blaZ*, *mecA*, and *APH(3’)-IIIa*, absent in other isolates.

**Table 2:** Antimicrobial resistance and resistance-associated gene content of institutional *S. argenteus* isolates.

Functional category	Gene	20250131/20250137*	20230185	20250208
Direct resistance	*fosB*	+	+	+
	*tet(38)*	+	+	+
Efflux pumps	*norA*	+	+	+
	*LmrS*	+	+	+
	*sdrM*	+	+	+
	*sepA*	+	+	+
	*norC*	+	+	+
Regulatory	*mgrA*	+	+	+
	*arlS*	+	+	+
	*arlR*	+	+	+
	*mepR*	+	+	+
	*mepA*	+	+	+
Acquired resistance	*blaZ*	−	−	+
	*mecA*	−	−	+
	*APH(3’)-IIIa*	−	−	+

Across the global dataset, the average number of AMR genes per strain was 14. Genes *tet(38)*, *arlR/S*, *mepA*/*R*, *mgrA*, *sdrM*, *norA/C*, and *LmrS* were detected in 100% of institutional isolates and >95% of global ST2250 genomes (197/207, 95.2% to 207/207, 100%; **Supplementary Table 4**).

**Figure 4:**
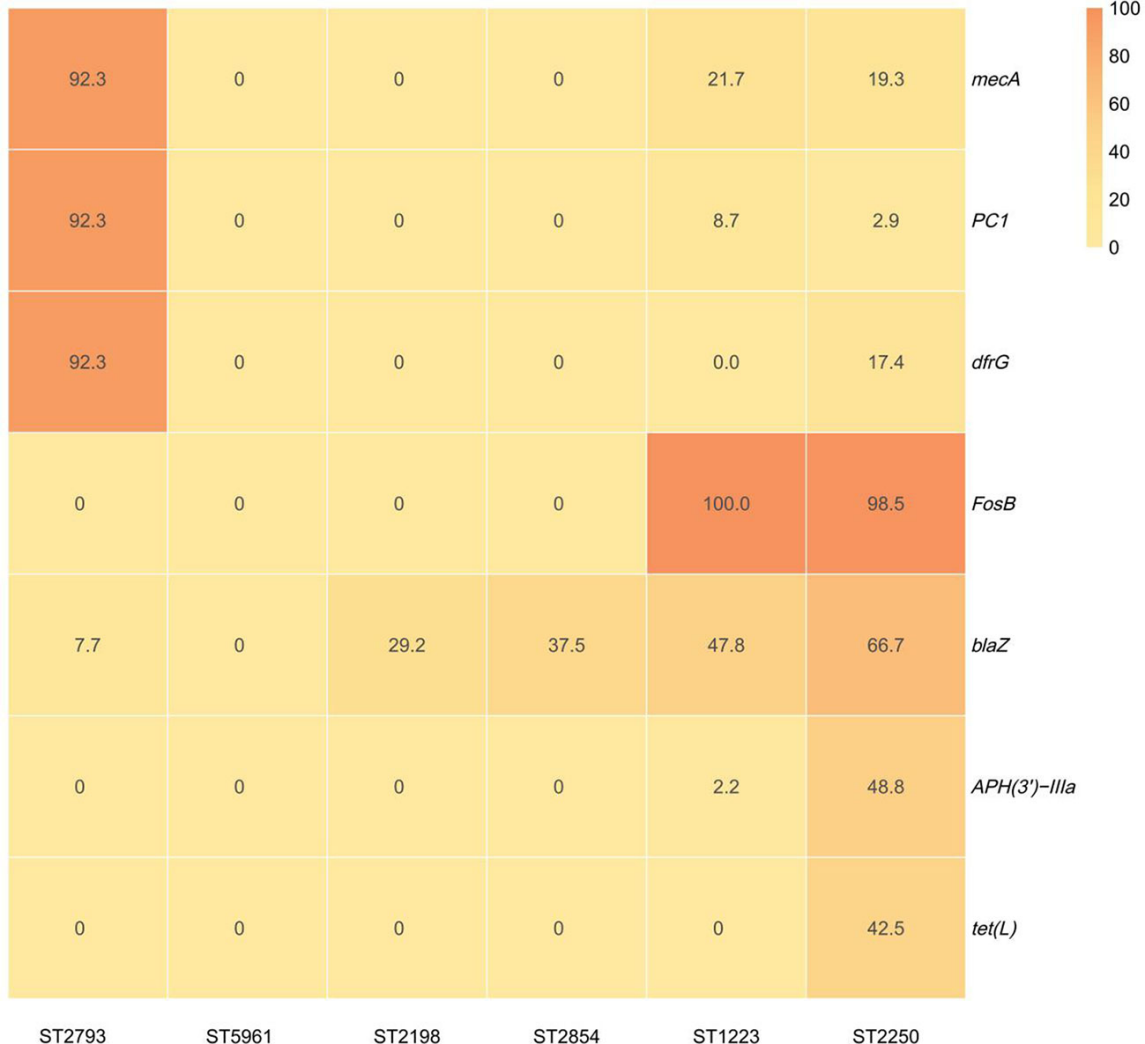
Antimicrobial resistance and resistance-associated gene profiles across *S. argenteus* sequence types.

Comparative analysis across sequence types revealed ST2250-specific resistance-associated signatures (**Figure 4**). ST2250 exhibited the highest prevalence of β-lactamase gene *blaZ* (66.7%) among all STs, markedly exceeding ST2198 (29.2%), ST2854 (37.5%), ST1223 (47.8%), and ST2793 (7.7%). The fosfomycin resistance gene *fosB* was nearly universal in ST2250 (98.5%) and ST1223 (100.0%), but completely absent in ST2793, ST5961, ST2198, and ST2854 (0%). Notably, ST2250 uniquely harbored two resistance-associated genes rarely detected in other STs: the tetracycline resistance gene *tet(L)* (42.5%) and the AMR gene *APH(3’)-IIIa* (48.8%). These genes were entirely absent in ST2793, ST5961, ST2198, and ST2854, with only ST1223 showing minimal *APH(3’)-IIIa* carriage (2.2%) and no *tet(L)* detection. ST2250 also demonstrated moderate methicillin resistance (*mecA*, 19.3%) and low trimethoprim resistance (*dfrG*, 17.4%). In contrast, ST2793 exhibited a distinct resistance pattern characterized by high *mecA* (92.3%), *PC1* (92.3%), and *dfrG* (92.3%) prevalence, but complete absence of *fosB*, *tet(L)*, and *APH(3’)-IIIa*. ST5961 carried no detectable resistance-associated genes among the seven analyzed, while ST2198 and ST2854 showed limited profiles with only *blaZ* detected.

Functional categories: (i) Direct resistance: genes encoding enzymes or efflux pumps that directly confer antibiotic resistance; (ii) Efflux pumps: resistance-associated efflux systems requiring induction for phenotypic expression; (iii) Regulatory: global regulators that modulate efflux pump expression but do not directly confer resistance. mgrA and arlS/arlR are included as CARD-annotated resistance-associated loci ^*^Isolates 20250131 (blood) and 20250137 (wound exudate) exhibited 0 core-genome SNP differences, confirming clonal identity and wound-to-bloodstream transmission. Isolate 20230185 was recovered in January 2023 from a distinct patient and differed by 198 SNPs from 20250131/20250137, representing an independent ST2250 lineage.

#### 3.5.2 Virulence genes

All four isolates harbored 56 conserved virulence genes. Five genes showed variability among the four institutional isolates: (1) 20230185 uniquely harbored *tsst-1* (toxic shock syndrome toxin-1); (2) 20250208 exclusively carried *sdrD* (serine-aspartate repeat protein); (3) *sdrE* was present in 20230185/20250208 but absent in 20250137; (4) *sak* (exoenzyme) and *scn* (immune modulation) were carried by 20230185, 20250131, and 20250137, but absent in 20250208; (5) *sdr* adhesin family variability: 20250208 had *sdrC/D/E*, 20250137 had *sdrC*, 20230185 had *sdrC/E* (**Supplementary Table 5**).

**Figure 5:**
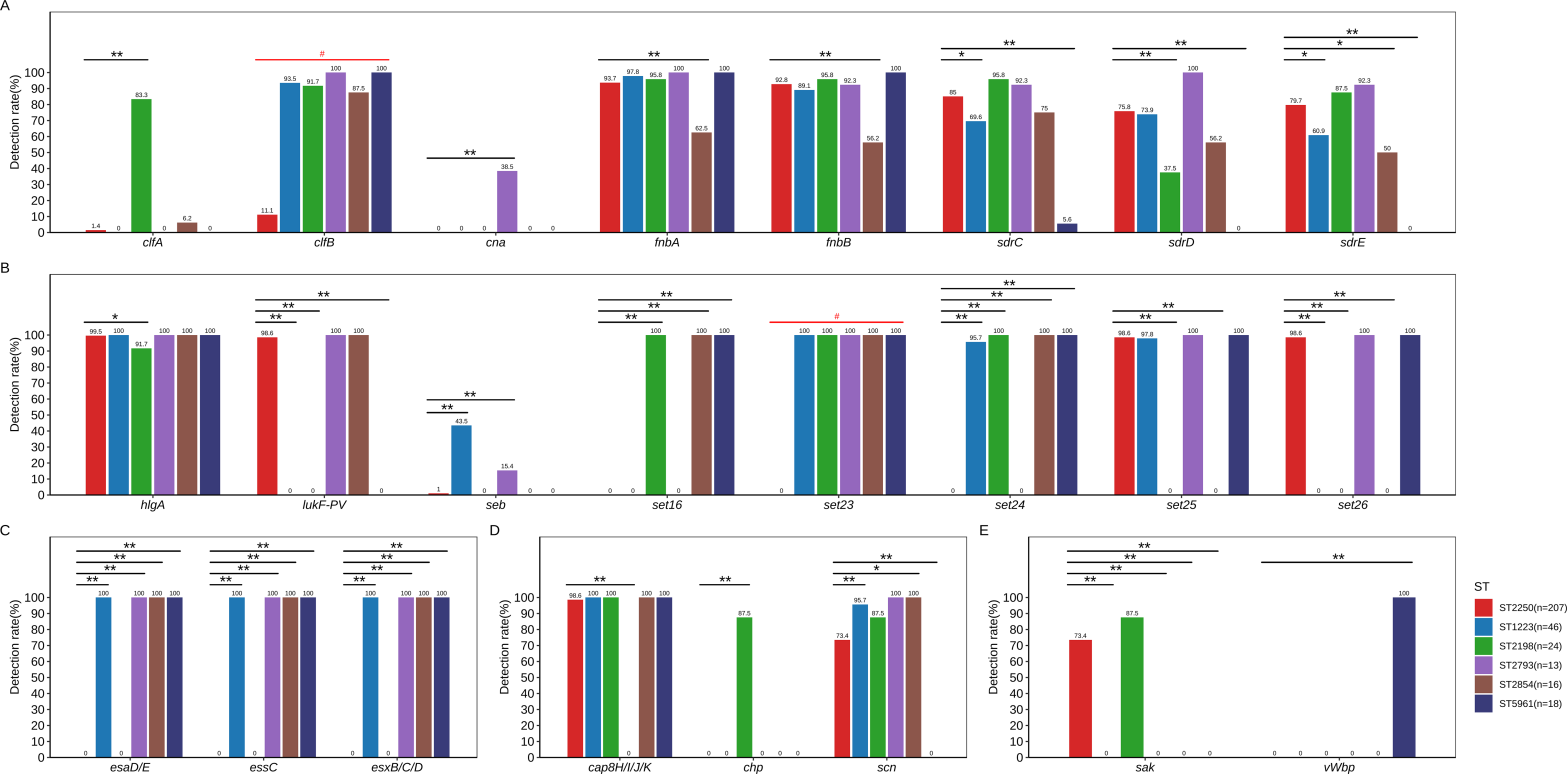
Comparative analysis of virulence genes in *S.* argenteus ST2250 versus other STs. **(A)** Adherence; **(B)** Exotoxin; **(C)** Effector delivery system; **(D)** Immune modulation; **(E)** Exoenzyme; Comparisons of virulence gene carriage rates between ST2250 (n=207) and other major STs (ST1223: n=46; ST2198: n=24; ST2793: n=26) were performed using Fisher’s exact test or chi-square test; *P<0.05, **P<0.01. Multiple comparisons were FDR-corrected (Benjamini-Hochberg method); #P<0.01 for ST2250 vs. all other STs combined.

The 370 global *S. argenteus* strains contain 85 virulence genes, including those related to adherence (*clf*, *fnb* and *sdr*), biofilm (*ica*), effector delivery system (*esa*, *ess* and *esx*), exoenzyme, exotoxin, immune modulation, motility, and nutritional/metabolic factor (**Supplementary Table 4**). A comparative analysis across major sequence types revealed significant heterogeneity in genes related to adherence, exotoxin, effector delivery systems, immune modulation, and exoenzymes (**Figure 5**). ST2250 exhibits a distinctive virulence gene profile characterized by significantly reduced carriage of *clfB* (11.1% vs. 87.5–100% in other STs, P<0.01) and complete absence of *set23* (0% vs. 100% in other STs, P<0.01).

ST2250 showed significantly lower detection rates for fibronectin-binding protein genes compared to other STs: *clfA* (1.4% vs. 83.3% in ST2198), *clfB* (11.1% vs. 93.5–100%). The collagen-binding protein gene *cna* was absent in ST2250 but present in 38.5% of ST2793 isolates. Among serine-aspartate repeat protein genes (**Figure 5A**), ST2250 exhibited intermediate prevalence: *sdrC* (85.0%), *sdrD* (75.8%), and *sdrE* (79.7%), significantly higher than ST5961 (0–5.6%, P<0.01) and ST1223 for *sdrC* (69.6%) and *sdrE* (60.9%, P<0.05).

Among enterotoxin factors (**Figure 5B**), ST2250 demonstrated near-universal carriage of gamma-hemolysin (*hlgA*, 99.5%) and Panton-Valentine leukocidin (*lukF-PV*, 98.6%), comparable to other STs. However, enterotoxin genes showed ST-specific patterns: *seb* was absent in ST2250 but detected in 43.5% of ST1223 and 15.4% of ST2793. Notably, ST2250 completely lacked the *set16/23/24* superantigen genes (0% prevalence), contrasting with their near-universal presence in ST2198, ST2854, and ST5961 (93.7–100%, P<0.05). Conversely, *set25* and *set26* were highly conserved in ST2250 (98.6% each, P<0.05) but absent in ST2198and ST2854.

The ESAT-6 secretion system components (*esaD/E*, *essC*, *esxB/C/D*) were significantly missing in ST2250 (0% prevalence), contrasting with their near-universal presence in ST1223, ST2793, ST2854, and ST5961 (100%); notably, ST2198 shared this absence pattern with ST2250 (**Figure 5C**). Conversely, superantigen genes *set25* and *set26* were highly conserved in ST2250 (98.6% each) but entirely absent in ST2198. Among other adhesion factors, ST2250 demonstrated markedly reduced carriage of fibronectin-binding protein *clfA* (1.4% vs. 83.3% in ST2198, P<0.01) while maintaining intermediate prevalence of serine-aspartate repeat proteins *sdrC* (85.0%), *sdrD* (75.8%), and *sdrE* (79.7%).

The cap8 polysaccharide capsule operon (*cap8H/I/J/K*) was highly conserved across ST2250 (98.6%) and other STs (97.8–100%), except ST2793, whereas the chemotaxis-inhibiting protein gene *chp* was absent in ST2250 but present in 87.5% of ST2198. Regarding immune evasion genes (**Figure 5D**), ST2250 exhibited a moderate prevalence of *scn* (73.4%) when compared to ST1223 (95.7%) and ST2793 (87.5%), and a lower carriage rate of *sak* (exoenzyme) (73.4% vs. 87.5% in ST2198, P < 0.05). The von Willebrand factor-binding protein gene *vWbp* was absent in all STs except ST5961 (100%, **Figure 5E**).

## 4 Discussion

This study provides definitive molecular evidence validating the diabetic foot ulcer as the source of bloodstream infection in a patient with septic shock. The zero core-genome SNP difference between blood and wound isolates confirms clonal identity, establishing a clear transmission trajectory from the foot lesion to systemic circulation. Comparative genomics of global isolates further revealed ST2250 as the dominant type (55.9%) with the majority of dated isolates reported during 2015-2019 (68.7%), featuring unique AMR (higher *APH(3’)-IIIa*/*tet(L)* and moderate *mecA*) and virulence profiles (reduced carriage of *clfB* and complete absence of *set23*) (Wang et al., 2016). These findings underscore ST2250’s pathogenic potential in high-risk populations like diabetic patients and its distinctive genomic and epidemiological features, with implications for clinical practice and surveillance.

The clinical presentation-uncontrolled type 2 diabetes with severe foot tissue infection progressing to septic shock and multiple organ dysfunction-underscores the pathogenic potential of ST2250 in high-risk populations. Diabetic patients are inherently susceptible to invasive *Staphylococcus* infections due to immunological impairment, neuropathy, and vascular insufficiency (Shettigar and Murali, 2020). This case highlights that *S. argenteus* ST2250 can induce life-threatening sepsis in this population even when pan-susceptible to antimicrobials, consistent with reports of severe invasive infections, such as joint infections (Jiang et al., 2018). Notably, *S. argenteus* was frequently misidentified as *S. aureus* due to phenotypic similarities (Zhang et al., 2016), which may lead to inappropriate management. This underscores the clinical value of advanced diagnostic tools such as MALDI-TOF MS with updated databases or WGS for accurate species identification, particularly in high-risk populations. A key genomic finding of this study is the complete absence of core-genome SNP differences between the blood (20250131) and wound (20250137) isolates. This zero-SNP divergence provides definitive molecular evidence that the two isolates are clonally identical, directly validating the diabetic foot ulcer as the primary infection focus and confirming hematogenous dissemination as the pathway for septic shock. Previous studies on *S. argenteus* bacteremia have often inferred infection sources based on clinical context alone, with limited molecular evidence to confirm the clonal homology between primary sites and bloodstream isolates (Jiang et al., 2018; Fang et al., 2024). In contrast, this study employed WGS-based core-genome SNP analysis to accurately trace the transmission chain in invasive *S. argenteus* infections. For clinical practice, this reinforces the importance of aggressive management of diabetic foot ulcers, including thorough debridement, targeted antimicrobial therapy, and wound care, as a critical measure to prevent local infection from progressing to life-threatening bacteremia. In community-acquired infections, cases of *S. argenteus* bacteremia were independently associated with a significantly higher mortality rate when compared to cases of methicillin - susceptible *S. aureus* bacteremia (Chen et al., 2018).

From a molecular epidemiological perspective, the study confirms ST2250 as the dominant sequence type of *S. argenteus* globally (55.9%), with a peak prevalence during 2015-2019 (68.7%). This temporal distribution likely reflects increased surveillance and research focus following the formal recognition of *S. argenteus* as a distinct species in 2015, rather than an exclusive surge in circulation (Tong et al., 2015). Notably, prior studies have shown that the clinical and epidemiological characteristics of *S. argenteus* exhibit regional variability. In Australia, S. argenteus (formerly CC75) was first described in indigenous communities and predominantly associated with skin and soft tissue infections (Ng et al., 2009). This finding is consistent with the results of this study, which suggest that *S. argenteus* ST6111 caused an outbreak involving skin or soft - tissue isolates in non - Asian countries. In contrast, in Thailand, *S. argenteus* has been recognized as an important cause of community - acquired invasive infections (Chantratita et al., 2016). Our findings align with these regional observations: ST2250 was highly prevalent among Thai isolates in the global dataset, and the report of ST2250-induced severe sepsis further supports its role as a significant pathogen of invasive disease in this region. Such variability may stem from regional differences in sequence type prevalence, host comorbidity profiles, antibiotic use patterns, and environmental reservoirs. Geographically, non-Asian countries in our study exhibited a polyclonal *S. argenteus* population (including ST2250, ST6111, and ST2793), which contrasts with the high prevalence of ST2250 in Thailand (Ruimy et al., 2009; Thaipadungpanit et al., 2015; Moradigaravand et al., 2017). Our institutional isolates further illustrate ST2250’s global adaptability: index case isolates (20250131/20250137) are closely related to 2012 Chinese strains (including a Shanghai pork isolate), aligning with ST2250’s high prevalence (74.6%) in Chinese retail foods (Wu et al., 2020) and supporting its dual niche colonization in clinical and food reservoirs. In contrast, the isolate 20230185 clusters with non-Asian clinical isolates (Italian blood, Brazilian foot secretion), and isolate 20250208 is genetically proximal to Thai isolates, confirming independent circulation and sporadic global dissemination of ST2250 lineages (Moradigaravand et al., 2017). Isolate 20230185 and isolate 20250208 differed by 198 and 212 SNPs, respectively, yielding an SNP accumulation rate of 8–9 per month. This rate is slightly higher than that reported for staphylococci: *S. argenteus* ST2250 exhibits a substitution rate of 4.66 mutations per genome per year in northeastern Thailand, comparable to the estimated 5.6 for a global *S. aureus* clonal complex (including ST121) (Kurt et al., 2013; Moradigaravand et al., 2017). Likely reflecting greater conservation of the core genome versus the whole genome, this temporal accumulation pattern further supports endemic circulation of ST2250 in our city rather than a point-source outbreak.

ST2250 exhibits a unique resistance profile characterized by the unique harborage of two resistance-associated genes rarely detected in other sequence types: the tetracycline resistance-associated gene *tet(L)* (42.5%) and the aminoglycoside resistance-associated gene *APH(3’)-IIIa* (48.8%). This ST-specific distribution pattern suggests either clonal founder effects or historical acquisition events unique to the ST2250 lineage, potentially through mobile genetic elements such as plasmids or transposons that subsequently stabilized within this clone (Moradigaravand et al., 2017; Chen et al., 2018). The *tet(L)* gene encodes an efflux pump conferring tetracycline resistance, while *APH(3’)-IIIa* mediates aminoglycoside resistance through 3’-phosphotransferase modification of antibiotic targets (Wang et al., 2021; Manoharan et al., 2022). The near-universal presence of *fosB* (98.6%)—a gene conferring resistance to fosfomycin, an agent used for skin/soft tissue infections and urinary tract infections-suggests empirical fosfomycin use may be ineffective for ST2250 infections, necessitating susceptibility testing (Monte and de Oliveira, 2024). High prevalence of *blaZ* (66.7%) and *APH(3’)-IIIa* (48.8%) rates indicate potential resistance to penicillins and aminoglycosides, respectively, while moderate *tet(L)* (42.5%) and *mecA* (19.3%) point to emerging tetracycline and methicillin resistance. Our updated epidemiological data show a modest increase in *mecA* carriage in ST2250 compared to the 14% reported in 2005–2018, while ST1223 exhibits a marked decrease in *mecA* prevalence (21.7% vs. 45%), and ST2793 maintains a high mecA rate (92.3%) (Goswami et al., 2021). Notably, *mecA* was not detected in the ST5961, ST2198, or ST2854 isolates from the dataset, which is in line with the previously reported sporadic distribution of methicillin resistance in these less prevalent sequence types (STs) (Giske et al., 2019). The high *blaZ* prevalence in ST2250 may be attributed to the chromosomal or plasmid origin of this gene, which is independent of sequence typing (Aung et al., 2021). All four institutional isolates exhibited pan-susceptibility to tested antibiotics despite harboring 12 putative AMR genes. This discrepancy reflects three factors: (i) functional gene classification—Only *fosB* and *tet(38)* encode direct resistance determinants; 10 genes are resistance-associated regulatory or efflux pump elements that require specific genetic backgrounds or environmental induction for phenotypic expression (Giske et al., 2019). *mgrA* and *arlS/arlR* are global regulators that modulate multidrug efflux pump expression but do not independently confer resistance (Fournier et al., 2001)—consistent with the observed pan-susceptibility despite their universal carriage. (ii) incomplete MIC panel coverage—the VITEK-2 panel did not include fosfomycin (*fosB* substrate) or tetracycline (*tet(38)* substrate), though tigecycline (a tetracycline derivative) was tested and all isolates were susceptible (MIC ≤0.12 μg/mL); and (iii) species-specific resistance expression—current AMR databases are curated from *S. aureus* and may not account for interspecies regulatory divergence (Chen et al., 2018; Giske et al., 2019; Wang et al., 2021; Monte and de Oliveira, 2024). Notably, *tet (38)* confers tetracycline resistance but does not necessarily confer tigecycline resistance, as the derivative’s bulky side chain may evade the Tet (38) efflux pump (Alfaiz, 2021; Wang et al., 2021). This illustrates the complexity of extrapolating *S. aureus* resistance mechanisms to *S. argenteus*. These findings demonstrate that genotype-based resistance prediction is currently unreliable in *S. argenteus*, and phenotypic susceptibility testing remains essential for clinical management.

ST2250 possesses a distinctive virulence gene profile characterized by significant gene losses and compensatory conservation of alternative virulence determinants. Most notably, ST2250 exhibits significantly reduced carriage of the fibronectin-binding protein gene *clfB* (11.1% vs. 87.5–100% in other STs, P<0.01) and complete absence of the superantigen-like toxin gene *set23* (0% vs. 100% in other STs, P<0.01). These features, combined with the complete absence of the ESAT-6 secretion system (*esaD/E*, *essC*, *esxB/C/D*), define a unique virulence architecture that distinguishes ST2250 from other *S. argenteus* lineages. Clumping factor B (*clfB*) promotes bacterial adherence to fibrinogen and keratinized epithelium, facilitating nasal colonization and skin infection (Alfaiz, 2021). The *set* genes encode staphylococcal exotoxin-like proteins with superantigenic properties that trigger massive cytokine release and toxic shock (Sato’o et al., 2021), while ST2198, ST2854, and ST5961 uniformly carry *set16/23/24* (93.7-100%). Meanwhile, the cap8 polysaccharide capsule operon (*cap8H/I/J/K*), *lukF-PV* (Panton-Valentine leukocidin), and *set25/26* (superantigens) are nearly conserved in ST2250. Intra-clonal virulence variability is also observed: the isolate 20230185 uniquely harbors *tsst-1* (toxic shock syndrome toxin-1), a gene rarely detected in food-derived or Japanese *S. argenteus* isolates, raising concerns about severe toxin-mediated complications (Kurt et al., 2013; Aung et al., 2017; Aung et al., 2019). Our findings are consistent with those of Hsu et al. (2020) on Taiwanese *S. argenteus* isolates: all strains harbored core virulence genes (e.g., hemolysin genes *hla* and *hld*), ~90% carried the immune evasion genes *scn* and *sak* (with a carriage rate of 75% in our cohort), and none contained specific toxin genes (e.g., *etb*, *edin*, *sea*, *sed*) (Hsu et al., 2020). High-prevalence genes (70–80%) including *sak*, *scn*, and *sdrC/D/E* further support its ability to evade host immunity and adhere to host tissues, while the rarity of *clfA* and *clfB* (<15%) suggests ST2250 relies on alternative adhesins for colonization. A notable discrepancy was that *lukF-PV* was universally present in our four isolates and in 65% of all 370 isolates (242/370), whereas it was not detected in Hsu et al.’s study cohort. PVL-positive *S. argenteus* has been linked to severe clinical manifestations (e.g., sepsis) and has also been documented in Thailand, Myanmar, France, and the United Arab Emirates (Dupieux et al., 2015; Chantratita et al., 2016; Aung et al., 2017; Monecke et al., 2024). This difference may reflect geographic subclone variation or discrepancies in detection methodologies.

This study has several inherent limitations. First, as a case report supplemented with institutional and global genomic analyses, the findings may not be generalizable to all *S. argenteus* ST2250 infections, particularly in diverse geographic or clinical settings. Second, the patient was discharged prematurely at the request of their family, precluding long-term outcome assessment and limiting our understanding of the clinical course of ST2250-induced sepsis. Third, the global genome dataset is potentially biased toward clinically relevant isolates, with under representation of environmental or food-derived strains, which may skew our interpretation of ST2250’s epidemiological characteristics. The temporal trends observed likely reflect expanded surveillance capacity and increased research attention following the 2015 species description (Tong et al., 2015), rather than true epidemiological expansion. Additionally, 102 genomes (27.6%) lacked collection year metadata, which may influence temporal interpretation. Fourth, functional validation of the identified AMR and virulence genes was not performed, so their clinical relevance remains to be confirmed. Finally, regional differences in sampling intensity and surveillance strategies may have influenced the observed geographic and temporal distribution of ST2250. Future studies should include larger, geographically diverse cohorts, functional assays to verify genomic findings, and longitudinal surveillance to better characterize the epidemiology and pathogenicity of ST2250.

## 5 Conclusions

This study confirms clonal transmission of *S. argenteus* ST2250 from diabetic foot ulcer to bloodstream through zero core-genome SNP identity between isolates. Comparative analysis of 370 global genomes showed ST2250 as the dominant sequence type (55.9%), with unique resistance and resistance-associated gene profiles including *tet(L)* (42.5%) and *APH(3’)-IIIa* (48.8%) rarely detected in other STs. ST2250 exhibited reduced *clfB* carriage (11.1%) and absence of ESAT-6 system components and *set23*, with compensatory conservation of *cap8* operon, *lukF-PV*, and *set25/26*. These findings provide molecular evidence for ST2250’s dissemination patterns and genomic characteristics in diabetic infections.

## Data Availability

The datasets presented in this study can be found in online repositories. The names of the repository/repositories and accession number(s) can be found in the article/**Supplementary Material**.

## Glossary

ANI: Average Nucleotide Identity
CARD: Comprehensive Antibiotic Resistance Database
cgMLST: Core Genome Multilocus Sequence Typing
CRISPR: Clustered Regularly Interspaced Short Palindromic Repeats
MALDI-TOF MS: Matrix-Assisted Laser Desorption/Ionization Time-of-Flight Mass Spectrometry
MSSA: Methicillin-Susceptible Staphylococcus aureus
MLST: Multilocus Sequence Typing
SNP: Single Nucleotide Polymorphism
VFDB: Virulence Factor Database
WGS: Whole-Genome Sequencing.
