# Supplementary material for: Staphylococcus argenteus ST2250 from diabetic foot sepsis: genomic insights into resistance and virulence
Source: Front Cell Infect Microbiol. 2026 Apr 29;16:1822021. doi: 10.3389/fcimb.2026.1822021 (PMC13167990; doi:10.3389/fcimb.2026.1822021)
Supplement: Supplementary file 1 [file Table1.docx]

**Supplementary Table S1** Top species abundance result statistics

| Name | TaxonomyID | 20230185 | 20250131 | 20250137 | 20250208 |
| --- | --- | --- | --- | --- | --- |
| *Staphylococcus argenteus* | 985002 | 92.089 | 89.565 | 91.396 | 92.074 |
| *Staphylococcus aureus* | 1280 | 7.267 | 9.788 | 7.99 | 7.249 |
| *Biseptimavirus IME136101* | 2846260 | 0.189 | 0.117 | 0.189 | 0 |
| *Staphylococcus schweitzeri* | 1654388 | 0.086 | 0.076 | 0.08 | 0.08 |
| *Staphylococcus sp. SM3655* | 2807625 | 0.027 | 0.044 | 0.055 | 0 |
| *Staphylococcus epidermidis* | 1282 | 0.016 | 0.018 | 0.013 | 0.042 |
| *Bacillus cereus* | 1396 | 0.018 | 0.025 | 0.011 | 0.022 |
| *Bacillus anthracis* | 1392 | 0.012 | 0.024 | 0.011 | 0.02 |

**Supplementary Table S2** Summary statistics of CheckM2 assembly results

| Sample name | Completeness_Model_Used | Completeness(%) | Contamination(%) |
| --- | --- | --- | --- |
| 20230185 | Neural Network (Specific Model) | 100 | 0.22 |
| 20250131 | Neural Network (Specific Model) | 100 | 0.66 |
| 20250137 | Neural Network (Specific Model) | 100 | 0.66 |
| 20250208 | Neural Network (Specific Model) | 100 | 0.15 |

**Supplementary Table S3** Temporal and geographic distribution of 370 global S. argenteus genomes

| Category | Subcategory | S. argenteus (N=370)n (%) | ST2250 (n=207)n (%) | ST2250 prevalence |
| --- | --- | --- | --- | --- |
|  | 2005-2009 | 6 (1.6%) | 2 (1%) | 33.3% |
| Time frame | 2010-2014 | 56 (15.1%) | 25 (12.1%) | 44.6% |
|  | 2015-2019 | 147 (39.7%) | 101 (48.8%) | 68.7% |
|  | 2020-2025 | 59 (15.1%) | 36 (17.4%) | 61.0% |
|  | Unknown | 102 (27.6%) | 43 (15.9%) | 35.8% |
| Geography | Non-Asian | 106 (28.6%) | 62 (16.8%) | 58.5% |
|  | Unknown | 80 (21.6%) | 29 (14.0%) | 36.6% |
|  | Thailand | 77 (20.8%) | 64 (23.7%) | 83.1% |
|  | China | 44 (11.9%) | 15 (5.6%) | 34.1% |
|  | Japan | 22 (5.9%) | 6 (2.2%) | 27.3% |

In Indonesia, Malaysia, Vietnam, Sri Lanka, South Korea, the United Arab Emirates, Singapore, India, and Israel, the total number of S. argenteus detected in these regions was less than 15 and was not included in the analysis.

**Supplementary Table S4** Virulence Gene Carriage Status of *S. argenteus* Across Major Sequence Types Globally and in This Study

| Virulence Gene Functional Category | Gene Name | Global ST2250 Carriage Rate (%)(n=207) | Carriage Status in 20250131/20250137/20230185/20250208  (+: Detected; -: Not Detected) | Carriage Rate in ST1223(n=46)/ST2198(n=24) |
| --- | --- | --- | --- | --- |
| Adherence | *clfA* | 1.5 | -/-/-/- | 0.0/83.3 |
|  | *clfB* | 11.1 | -/-/-/- | 93.5/91.7 |
|  | *cna* | 0 | -/-/-/- | 0.0/0.0 |
|  | *fnbA* | 93.7 | +/+/+/+ | 97.8/95.8 |
|  | *fnbB* | 92.8 | +/+/+/+ | 89.1/95.8 |
|  | *sdrC* | 85 | +/+/+/+ | 69.6/95.8 |
|  | *sdrD* | 75.9 | -/-/-/+ | 73.9/37.5 |
|  | *sdrE* | 79.7 | -/-/+/+ | 60.9/87.5 |
| Biofilm | *icaA* | 100 | +/+/+/+ | 100.0/100.0 |
|  | *icaB* | 100 | +/+/+/+ | 100.0/100.0 |
|  | *icaC* | 100 | +/+/+/+ | 100.0/100.0 |
|  | *icaD* | 100 | +/+/+/+ | 100.0/100.0 |
|  | *icaR* | 100 | +/+/+/+ | 100.0/100.0 |
| Effector delivery system | *esaA* | 100 | +/+/+/+ | 100.0/100.0 |
|  | *esaB* | 100 | +/+/+/+ | 100.0/100.0 |
|  | *esaD* | 0 | -/-/-/- | 100.0/0.0 |
|  | *esaE* | 0 | -/-/-/- | 100.0/0.0 |
|  | *esaG* | 99.5 | +/+/+/+ | 100.0/100.0 |
|  | *essA* | 100 | +/+/+/+ | 100.0/100.0 |
|  | *essB* | 100 | +/+/+/+ | 100.0/100.0 |
|  | *essC* | 0 | -/-/-/- | 100.0/0.0 |
|  | *esxA* | 100 | +/+/+/+ | 100.0/100.0 |
|  | *esxB* | 0 | -/-/-/- | 100.0/0.0 |
|  | *esxC* | 0 | -/-/-/- | 100.0/0.0 |
|  | *esxD* | 0 | -/-/-/- | 100.0/0.0 |
| Exoenzyme | *aur* | 100 | +/+/+/+ | 100.0/100.0 |
|  | *eta* | 0 | -/-/-/- | 0.0/4.2 |
|  | *hysA* | 98.6 | +/+/+/+ | 100.0/100.0 |
|  | *lip* | 100 | +/+/+/+ | 100.0/100.0 |
|  | *sak* | 73.4 | +/+/+/- | 0.0/87.5 |
|  | *sspA* | 100 | +/+/+/+ | 100.0/100.0 |
|  | *sspB* | 100 | +/+/+/+ | 100.0/100.0 |
|  | *sspC* | 100 | +/+/+/+ | 100.0/100.0 |
|  | *vWbp* | 0 | -/-/-/- | 0.0/0.0 |
| Exotoxin | *hlb* | 100 | +/+/+/+ | 100.0/100.0 |
|  | *hld* | 100 | +/+/+/+ | 100.0/100.0 |
|  | *hlgA* | 99.5 | +/+/+/+ | 100.0/91.7 |
|  | *hlgB* | 100 | +/+/+/+ | 100.0/100.0 |
|  | *hlgC* | 100 | +/+/+/+ | 100.0/100.0 |
|  | *hly/hla* | 100 | +/+/+/+ | 100.0/100.0 |
|  | *lukF-PV* | 98.6 | +/+/+/+ | 0.0/0.0 |
|  | *lukS-PV* | 6.3 | -/-/-/- | 0.0/0.0 |
|  | *seb* | 1 | -/-/-/- | 43.5/0.0 |
|  | *sec* | 1 | -/-/-/- | 0.0/0.0 |
|  | *selk* | 2.4 | -/-/-/- | 0.0/0.0 |
|  | *sell* | 1 | -/-/-/- | 0.0/0.0 |
|  | *selq* | 2.4 | -/-/-/- | 0.0/0.0 |
|  | *set16* | 0 | -/-/-/- | 0.0/100.0 |
|  | *set17* | 100 | +/+/+/+ | 97.8/100.0 |
|  | *set20* | 100 | +/+/+/+ | 100.0/100.0 |
|  | *set22* | 100 | +/+/+/+ | 100.0/100.0 |
|  | *set23* | 0 | -/-/-/- | 100.0/100.0 |
|  | *set24* | 0 | -/-/-/- | 95.7/100.0 |
|  | *set25* | 98.6 | +/+/+/+ | 97.8/0.0 |
|  | *set26* | 98.6 | +/+/+/+ | 0.0/0.0 |
|  | *tsst-1* | 1.5 | -/-/+/- | 0.0/0.0 |
| Immune modulation | *adsA* | 100 | +/+/+/+ | 100.0/100.0 |
|  | *cap8B* | 100 | +/+/+/+ | 100.0/100.0 |
|  | *cap8C* | 100 | +/+/+/+ | 100.0/100.0 |
|  | *cap8D* | 100 | +/+/+/+ | 100.0/100.0 |
|  | *cap8E* | 100 | +/+/+/+ | 100.0/100.0 |
|  | *cap8F* | 100 | +/+/+/+ | 100.0/100.0 |
|  | *cap8G* | 100 | +/+/+/+ | 100.0/100.0 |
|  | *cap8H* | 98.6 | +/+/+/+ | 100.0/100.0 |
|  | *cap8I* | 100 | +/+/+/+ | 100.0/100.0 |
|  | *cap8J* | 99.5 | +/+/+/+ | 100.0/100.0 |
|  | *cap8K* | 99.5 | +/+/+/+ | 100.0/100.0 |
|  | *cap8L* | 100 | +/+/+/+ | 100.0/100.0 |
|  | *cap8M* | 100 | +/+/+/+ | 100.0/100.0 |
|  | *cap8O* | 100 | +/+/+/+ | 100.0/100.0 |
|  | *cap8P* | 100 | +/+/+/+ | 100.0/100.0 |
|  | *capA* | 100 | +/+/+/+ | 100.0/100.0 |
|  | *capN* | 100 | +/+/+/+ | 100.0/100.0 |
|  | *chp* | 0 | -/-/-/- | 0.0/87.5 |
|  | *sbi* | 100 | +/+/+/+ | 100.0/100.0 |
|  | *scn* | 73.4 | +/+/+/- | 95.7/87.5 |
| Motility | *fliE* | 0.5 | -/-/-/- | 0.0/0.0 |
| Nutritional/Metabolic factor | *isdC* | 100 | +/+/+/+ | 100.0/100.0 |
|  | *isdD* | 100 | +/+/+/+ | 100.0/100.0 |
|  | *isdE* | 100 | +/+/+/+ | 100.0/100.0 |
|  | *isdG* | 100 | +/+/+/+ | 100.0/100.0 |
|  | *isdI* | 100 | +/+/+/+ | 100.0/100.0 |
|  | *srtB* | 99.5 | +/+/+/+ | 100.0/100.0 |

Notes: 1. Carriage rate was calculated as “number of isolates carrying the gene/total number of isolates in the ST × 100%”; 2. A total of 207 ST2250 isolates were included in the global dataset, with 44 ST1223 isolates (mainly food-derived) and 26 ST2793 isolates (mainly from clinical invasive infections); 3. The coa gene was only detected in ST5057 (n=1) and samples with unknown ST; seh was only found in samples with unknown ST.

**Supplementary Table S5** Distribution of variable virulence genes among *S. argenteus* ST2250 isolates

| **Gene** | **Function** | **20230185** | **20250137/20250131** | **20250208** |
| --- | --- | --- | --- | --- |
| **tsst-1** | Toxic shock syndrome toxin-1 (superantigen) | **+** | - | - |
| **sdrD** | Serine-aspartate repeat protein D (adhesin) | - | - | **+** |
| sdrE | Serine-aspartate repeat protein E (adhesin) | + | - | + |
| sak | Staphylokinase (immune evasion) | + | + | - |
| scn | Staphylococcal complement inhibitor (immune evasion) | + | + | - |

Isolates 20250131 (blood) and 20250137 (wound exudate) are clonally identical with 0 core-genome SNP differences and identical virulence gene profiles. Data shown represent both isolates.

**Supplementary Figure S1** Heatmap of ANI values among sample genomes


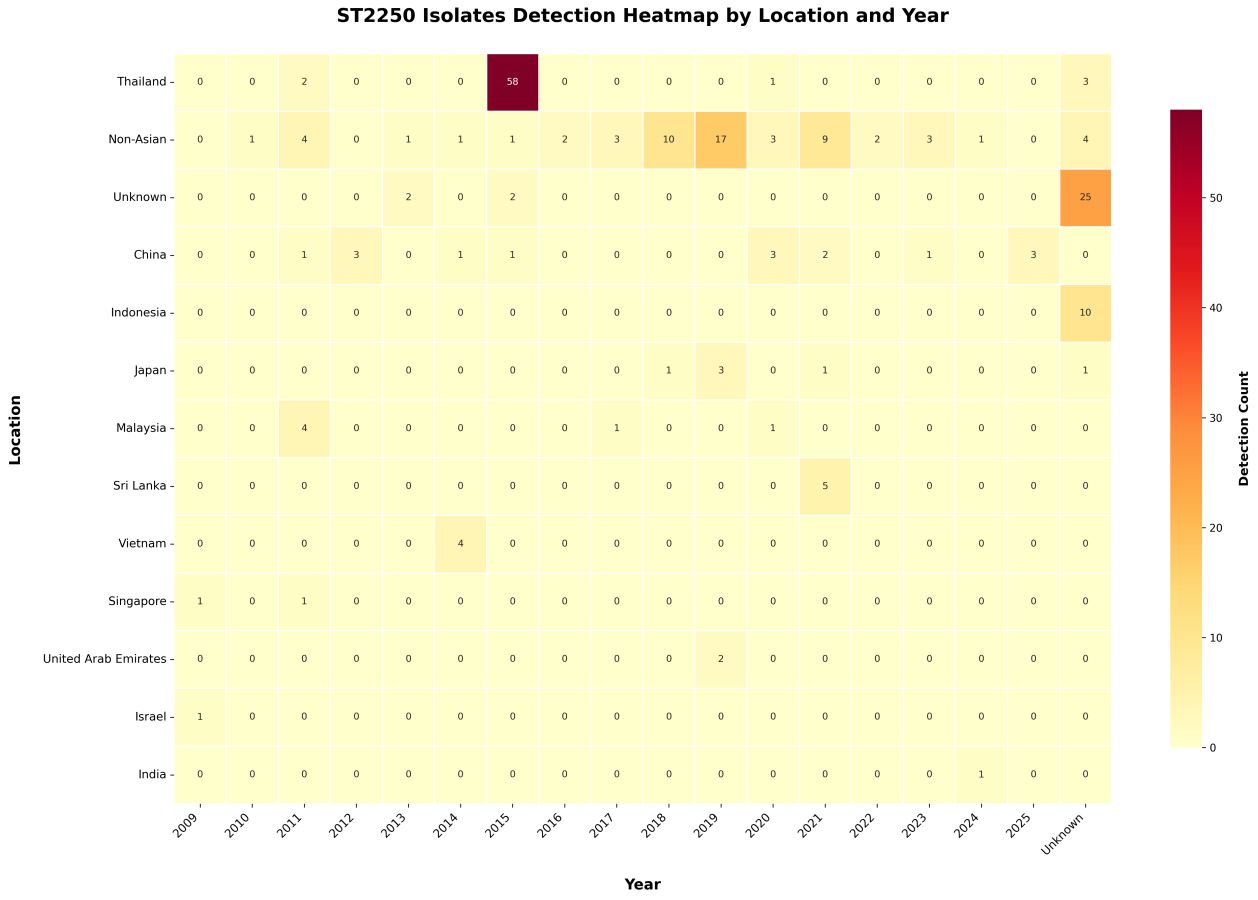


**Supplementary Figure S2** ST2250 Isolates Detection Heatmap by Location and Year. The heatmap displays the count of ST2250 isolates across geographic locations (rows) and collection years (columns), based on our analysis of 370 global *S. argenteus* genomes. Color intensity indicates detection count, with dark red representing the highest concentration (n=58, Thailand 2015). The "Unknown" column represents isolates with missing collection year metadata.
